# A rare case of sclerosing angiomatoid nodular transformation of spleen: A case report

**DOI:** 10.1016/j.ijscr.2024.110745

**Published:** 2024-12-15

**Authors:** Seifu Alemu, Bilisuma Mulatu, Abdo Kedir, Milkias Minka, Workneh Tesfaye, Wondu Reta Demissie

**Affiliations:** a Jimma University, Institute of Health, Faculty of Medical Sciences, Department of Surgery, Jimma, Ethiopia; b Jimma University, Institute of Health, Faculty of Medical Sciences, Department of Pathology, Jimma, Ethiopia; c Jimma University, Institute of Health, Faculty of Medical Sciences, Department of Biomedical Sciences, Jimma, Ethiopia

**Keywords:** Sclerosing angiomatoid nodular transformation of spleen, Surgical intervention, Case report

## Abstract

**Introduction and importance:**

Sclerosing angiomatoid nodular transformation of the spleen is a rare benign vascular lesion arising from red pulp of spleen with unknown etiopathogenesis. It is a non-neoplastic condition that affects the spleen only; not described in other sites except one case reported in adrenal gland. Epidemiologically it has slight female predilection. It is a very rare ailment where the present finding is very crucial in the management of similar cases so far.

**Case presentation:**

A 31 years old female patient presented with left flank pain and constipation of 01 year duration. An abdominopelvic CT scan showed a hypodense mass in the left upper quadrant of abdomen just anterior to inferior pole of spleen with the conclusion of mesenteric mass likely gastrointestinal stromal tumor. Finally, it was decided and exploratory laparotomy was done and the surgeon identified the mass in the inferior pole of spleen and total splenectomy was done, and the specimen is sent for pathologic evaluation. The final histopathologic diagnosis became sclerosing angiomatoid nodular transformation of spleen with the classic microscopic findings of multiple confluent angiomatoid nodules surrounded by variable concentric fibrosclerotic stroma.

**Clinical discussion:**

Sclerosing angiomatoid nodular transformation of spleen is a benign incidentally identified vascular condition of red pulp in the majority of cases. Preoperative diagnosis is not easy since it is difficult to obtain the tissue from the spleen for pathological study. Recently, splenectomy has become the more standard procedure for the spleen for both diagnosis and treatment with no recurrence reported after splenectomy.

## Highlights

• Sclerosing angiomatoid nodular transformation of the spleen is a rare benign vascular lesion arising from red pulp of spleen with unknown etiopathogenesis.
• It is a non-neoplastic condition that affects the spleen only; not described in other sites except one case reported in adrenal gland.
• It is a very rare ailment where the present finding is very crucial in the management of similar cases so far.
• It requires multimodal diagnostic modalities.
• Total splenectomy is recommedended to avoid recurrence.

## 1 Introduction

Sclerosing angiomatoid nodular transformation (SANT) of the spleen is a rare benign vascular lesion with unknown etiopathogenesis and with definite features of imaging, histopathology and immunohistochemistry [1,2]. It is a non-neoplastic condition that affects the spleen only; not described in other sites except one case reported in adrenal gland. Commonly affected age groups are 30–60 years old with mean age of 48 years and it shows slight female predilection with female to male ratio of 2:1. Majority of the cases are asymptomatic and SANT is commonly discovered incidentally by routine imaging studies and at the time of surgery for unrelated conditions [[3], [4], [5]]. It was first described by Martel et al. in 2004, and to date, only 258 cases have been reported in literatures [6].

However, the real incidence and prevalence are still unknown because all SANT of spleen cases are not reported. In the current case report, we thoroughly narrated a case of SANT of spleen managed in our hospital by rigorous review of the literatures.

## 2 Case presentation

A 31 years old female patient presented to Firomsis General Hospital in Jimma city, South West Ethiopia with the complaints of left flank pain and constipation of 01 year duration. Her physical examination findings showed a stable vital signs of BP = 110/70 mmHg, PR = 90 bpm, SPO2 = 97 % and abdominal examination showed slightly distended and doughy abdomen with no other pertinent findings. Abdominal CT scan showed there is a hypodense mass in the left upper quadrant of abdomen just anterior to inferior pole of spleen with enhancing heterogeneous surface and smooth border measuring 6.3 × 5.7 × 5 cm and the impression was left upper quadrant enhancing well defined mesenteric mass likely GIST. Then, the patient was scheduled for exploratory laparotomy and resection of mass. An informed and written consent was taken and laparotomy was done under general anesthesia.

### 2.1 Surgical procedure

The patient was put in supine position, the skin was cleaned and the abdominal cavity was entered through midline surgical incision. An intraoperative finding showed a huge spleen with a mass arising from the lower pole of spleen. No other pathology identified. Finally, a total splenectomy was done, hemostasis was secured and after correct instruments and gauze count made, the abdomen was closed in layers, and the specimen was sent to department of pathology for histopathologic examination.

### 2.2 Pathologic findings

#### 2.2.1 Macroscopic/gross description

The size of spleen was 18.5 × 8 × 6cm and there was a single well circumscribed solid mass with smooth surface measuring 6 × 5.4 × 4 cm in the splenic lower pole. Cut section through the mass shows slightly well delineated firm mass which is grayish to white-tan in color with large central stellate scar measuring about 6 × 5.4 × 4 cm. Cut section through adjacent spleen is unremarkable.

#### 2.2.2 Microscopic description

Section shows an unencapsulated but well circumscribed mass composed of multiple confluent angiomatoid nodules surrounded by variable concentric fibrosclerotic stroma. The nodules contain variable sized blood vessels with extravasated red blood cells. The periphery of the nodules show abundant collagen fibers infiltrated by few plasma cells and lymphocytes. Adjacent splenic tissue shows normal histology of spleen.

### 2.3 Post-operative outcome

The patient started sips on the postoperative of 48 h and soft diet on the following day. She was discharged on postoperative day of 5 days without any remarkable events. She was advised on danger signs and discharged with Amoxicillin 1 g PO daily for 03 months with proton pump inhibitors for appointment.

## 3 Discussion and conclusion

SANT of the spleen is a rare benign vascular lesion with unknown etiopathogenesis and with definite features of imaging, histopathology and immunohistochemistry [3,4,7]. It is a non-neoplastic condition that affects the spleen only; not described in other sites except one case reported in adrenal gland. Majority of the cases are asymptomatic and SANT is commonly discovered incidentally by routine imaging studies and at the time of surgery for unrelated conditions. The splenic lesion was solitary, measuring 3 to 17 cm and sharply demarcated from the surrounding parenchyma [1,2,5,[8], [9], [10], [11]]. It was first described by Martel et al. in 2004 [6].

The pathogenesis of SANT is unclear. Martel et al. postulated that SANT was due to stromal proliferation and the inter-nodular zones were similar to inflammatory pseudotumor. So, SANT may represent a hamartomatous transformation of red pulp of spleen in response to an exaggerated non-neoplastic stromal proliferation. But in majority of cases, the etiology is unknown [12,13].

Most of cases are asymptomatic and discovered accidentally, usually on imaging study. On imaging study, they present as solitary, round, lobulated mass which is centrally hypodense with peripheral enhancing portions. Contrast CT/MRI scan shows heterogeneously hypo-enhancing lesion during arterial and venous phase with an early peripheral-enhancing radiating lines and delayed enhancement of the central area due to fibrous tissue. So, these radiologic findings may be suggestive of benign vascular lesions such as hemangiomas; however, they can also be malignant vascular tumors such as angiosarcomas. Therefore, it is important to definitively characterize these lesions by histopathology [3,4,14].

**Fig. 1:**
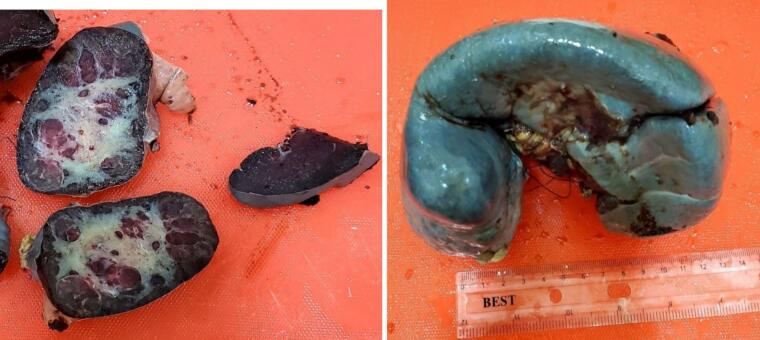
Gross examination shows slightly well delineated mass which is grayish to white-tan in color with large central stellate scar.

On gross examination, SANT is usually a solitary, well-circumscribed nodule that is distinct from the surrounding splenic parenchyma. Histologically, it has a vaguely nodular architecture surrounded by a hyaline shell, with markedly cellular blood vessels similar to those seen in hemangiomas. Its stroma consists of myxoid and sclerotic fibrous tissue with some myofibroblasts, lymphocytes, plasma cells and hemosiderin-containing macrophages. Immunohistochemical analysis has suggested SANT to be a predominantly polyclonal reactive lesion, lacking evidence of any relationship with IgG4-related sclerosing lesions, or EBV-positive stromal cells [15] (Fig. 1).

SANT has traditionally demonstrated three distinct patterns of immune staining: CD34+/CD31+/CD8−, CD34−/CD31+/CD8+ and CD34−/CD31+/CD8−, which indicates derivation from capillaries, splenic sinusoidal lining cells, or small veins, respectively. Early lesions have also demonstrated perivascular concentric fibrosis, as seen in our case [16].

Sometimes fine-needle biopsy from the spleen has been used to ascertain tissue diagnosis. However, this procedure has some problems with bleeding or tumor dissemination. Therefore, in most cases, splenectomy is recommended for diagnosis and treatment with no reported recurrence after resection [17].

Splenic biopsy has been employed and suggested as a good core biopsy can be used to distinguish SANT from other lesions. However, there is a worry about risk of intra peritoneal seeding if the lesion biopsied proves to be angiosarcoma and other complications such as splenic rupture and bleeding are noted with splenic biopsy [2,[18], [19], [20]].

**Fig. 2:**
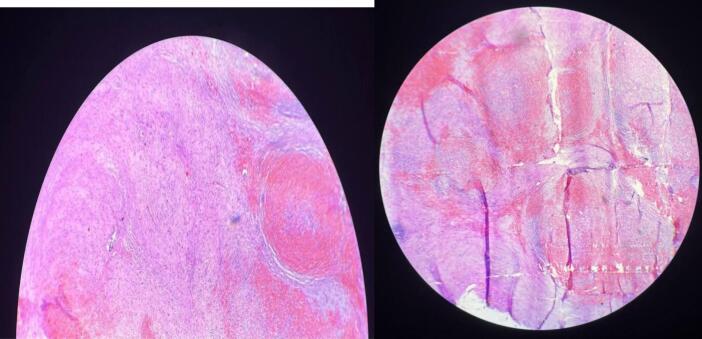
Microscopic examination shows multiple confluent angiomatoid nodules surrounded by variable concentric fibrosclerotic stroma.

It is difficult to rule out benign pathological conditions of the spleen such as inflammatory pseudotumor or hamartoma and also nodular carcinomatous metastasis to spleen by using imaging modalities alone. Therefore, for the diagnosis of SANT of spleen clinical history, radiological modalities and histopathological examinations with the immunohistochemistry markers of blood vessels together are very important. Overall, SANT has good prognosis after total splenectomy with no risk of recurrence (Fig. 2).

## CRediT authorship contribution statement

SA, BM, AK contributed substantially from the patient evaluation to writing up of the manuscript; MM, WT and WRD contributed in revision of the paper.

## Consent

Written informed consent was obtained from the mother for publication of this case report and accompanying images. A copy of the written consent is available for review by the Editor-in-Chief of this journal on request.

## Ethical approval

Ethical approval for this study was provided by the Ethical Committee of the hospital on 15 September 2024 (IRB/FPH/67/2024).

## Guarantor

Seifu Alemu, Bilisuma Mulatu and Abdo Kedir will take the primary responsibility of the study.

## Provenance and peer review

Not commissioned, externally peer-review.

## Funding

This work does not received any funds.

## Declaration of competing interest

The authors declare that they have no competing interests.
